# Life cycle inventory data for ethyl levulinate production from Colombian rice straw

**DOI:** 10.1016/j.dib.2022.108681

**Published:** 2022-10-20

**Authors:** Cristhian Cañon, Nestor Sanchez, Martha Cobo

**Affiliations:** Energy, Materials and Environment Laboratory, Faculty of Engineering, Universidad de La Sabana, Campus Universitario Puente del Común, Km. 7 Autopista Norte, Bogotá, Colombia

**Keywords:** Biomass, Biorefinery, Economic analysis, Life cycle assessment, Water consumption

## Abstract

This data article is associated with the research article “Sustainable production of ethyl levulinate by levulinic acid esterification obtained from Colombian rice straw”. This paper shows the methodology to calculate the Life Cycle Inventory (LCI) of the foreground system to perform the Life Cycle Assessment (LCA) of the ethyl levulinate (EL) production from Colombian rice straw (RS). This process encompasses two main stages: (i) RS production (involving cultivation and harvesting) and (ii) EL production (involving acid hydrolysis, levulinic acid (LA) purification, and El production). On one hand, foreground data related to paddy rice cultivation was gathered from the literature review. Besides, emissions of the cultivation stage were calculated using the IPCC (Intergovernmental Panel on Climate Change) methodology. The SQCB (Sustainable Quick Check for Biofuels) methodology was used to calculate NH_3_, NO_x_, N_2_O and NO_3_ emissions, whereas the SALCA (Swiss Agricultural Life Cycle Assessment) model was used to calculate phosphorous emissions to water. The Turc method was employed to calculate the irrigation requirements based on the rainfall and agrological features of rice culture. On the other hand, foreground data related to RS conversion to EL within a biorefinery scheme was obtained from simulation using Aspen Plus v.12. Lastly, background data associated with raw materials, catalysts, and utilities were gathered from Ecoinvent database. All the inventories are meaningful to carry out future environmental assessments involving sustainable production processes using RS as raw material or biorefinery processes using dilute acid hydrolysis.

**Specifications Table**

**Table:** 

Subject	Chemical Engineering: Process Chemistry and Technology
Specific subject area	Life Cycle Inventory and Chemical Process Simulation
Type of data	Table and Figure
How data were acquired	The foreground data was acquired from process simulation using Aspen Plus v.12 (Aspen Tech, MA, USA) and Aspen Economic Analyzer v.12 (Aspen Tech, MA, USA). The background data was gathered from Ecoinvent v.3.4 and the literature review for data related to rice cultivation.
Data format	Raw and processed
Description of data collection	Primary data concerning mass and energy balance to produce ethyl levulinate from Colombian rice straw was obtained from the literature review, Aspen Plus simulations, databases such as Ecoinvent version 3.4, scientific reports and academic theses. Data for paddy rice cultivation was obtained from literature review and calculations using methods which are described along this document.
Data source location	Institution: Universidad de La Sabana City/Town/Region: Chía, Cundinamarca Country: Colombia
Data accessibility	Raw data Repository name: Ethyl_levulinate_from_Colombian_Rice_Straw DOI: 10.17632/p4prb8mb32.1
Related research article	C. Cañon, N. Sanchez, M. Cobo, Sustainable production of ethyl levulinate by levulinic acid esterification obtained from Colombian rice straw, J. Clean. Prod., 377 (2022) 134276. https://doi.org/10.1016/j.jclepro.2022.134276

**Value of the Data**

• The data shown in this contribution support the Life Cycle Assessment depicted in the main article. This manuscript presents information that were not included in a explicit way in the related research article such as details of the process simulations performed, sensitivity analysis for distillation columns, paddy rice culture inventory calculation, and contribution analysis impact categories assessed.
• The data shown in this document could be used by anyone who wants to assess the environmental performance of the e-fuels and bioproducts production derived from Colombian rice straw.
• These data could be used as input in other Life Cycle Assessment studies and for simulate similar processes such as the production of other bio-products (e.g., gamma-valerolactone, furfuryl alcohol, furanic compounds, other levulinate esters, among others) derived from rice straw, and other Life Cycle Assessments that consider rice straw as raw material.

## 1 Objective

The data presented in this data article is generated as a result of the techno-economic and environmental assessment performed to evaluate the preliminary feasibility for the production of ethyl levulinate from Colombian rice straw. This data is mainly focused on two aspects. First, the description of the simulation process performed in Aspen Plus v12 (AspenTech, Bedford, MA, USA) as well as the mass and energy balance obtained for the overall process. Second, the life cycle inventory obtained from the simulation process and the literature review for the paddy rice cultivation in Colombian context and the subsequent biorefinery process. This Life Cycle Assessment was performed in OpenLCA v1.10 (GreenDelta, Berlin, Germany). Economic data for the process economic assessment is included in this data article. The detailed life cycle inventories presented in this article support the main article discussion about environmental impacts related to the production of ethyl levulinate from rice straw and the identification of improvement opportunities for the process under study. Likewise, this inventory data could be used for further research related to the valorization of agro-industrial residues in the Colombian context.

## 2 Data Description

**Fig. 1:**
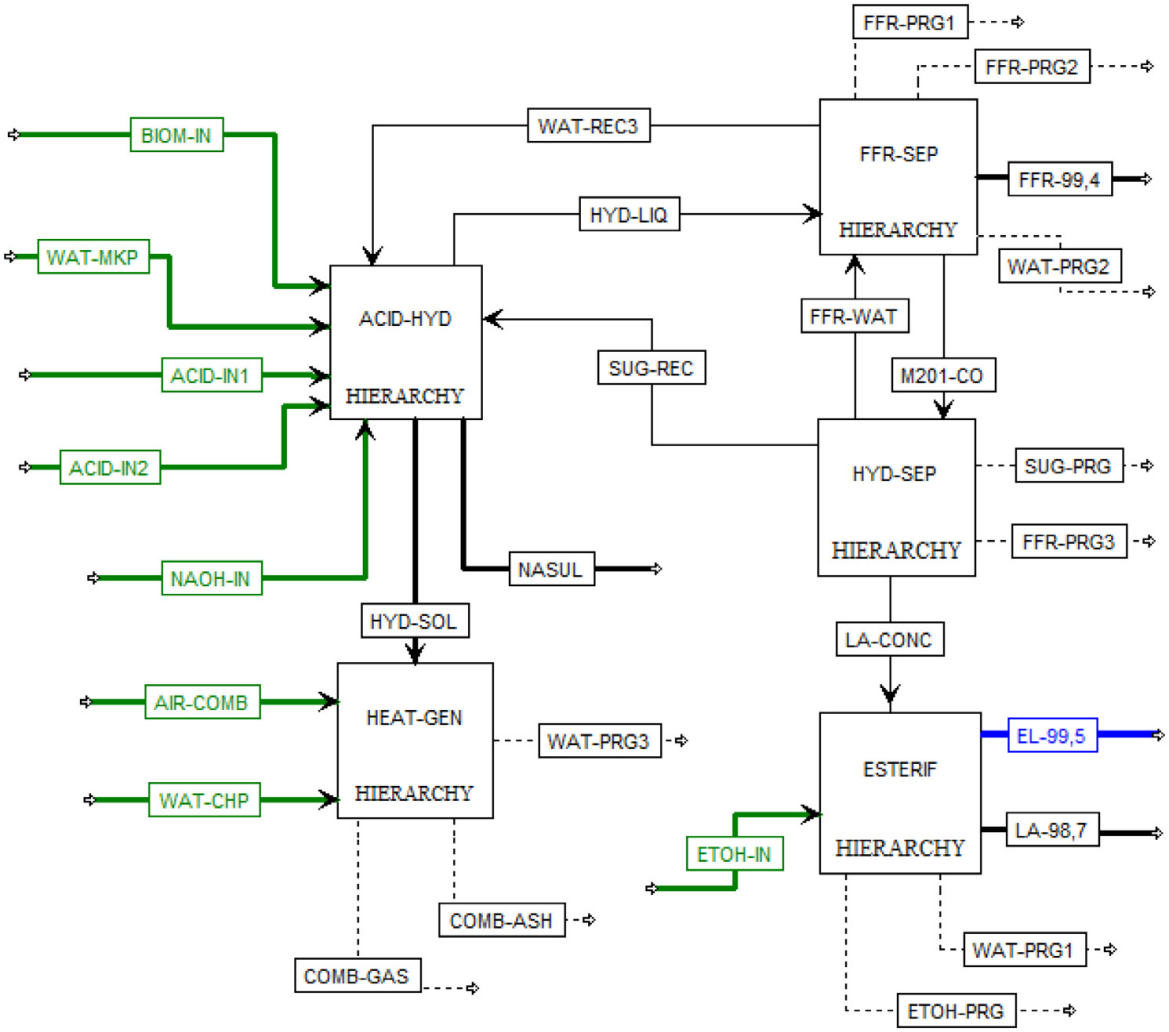
Simulation process in Aspen Plus (alternative scenario).

**Fig. 2:**
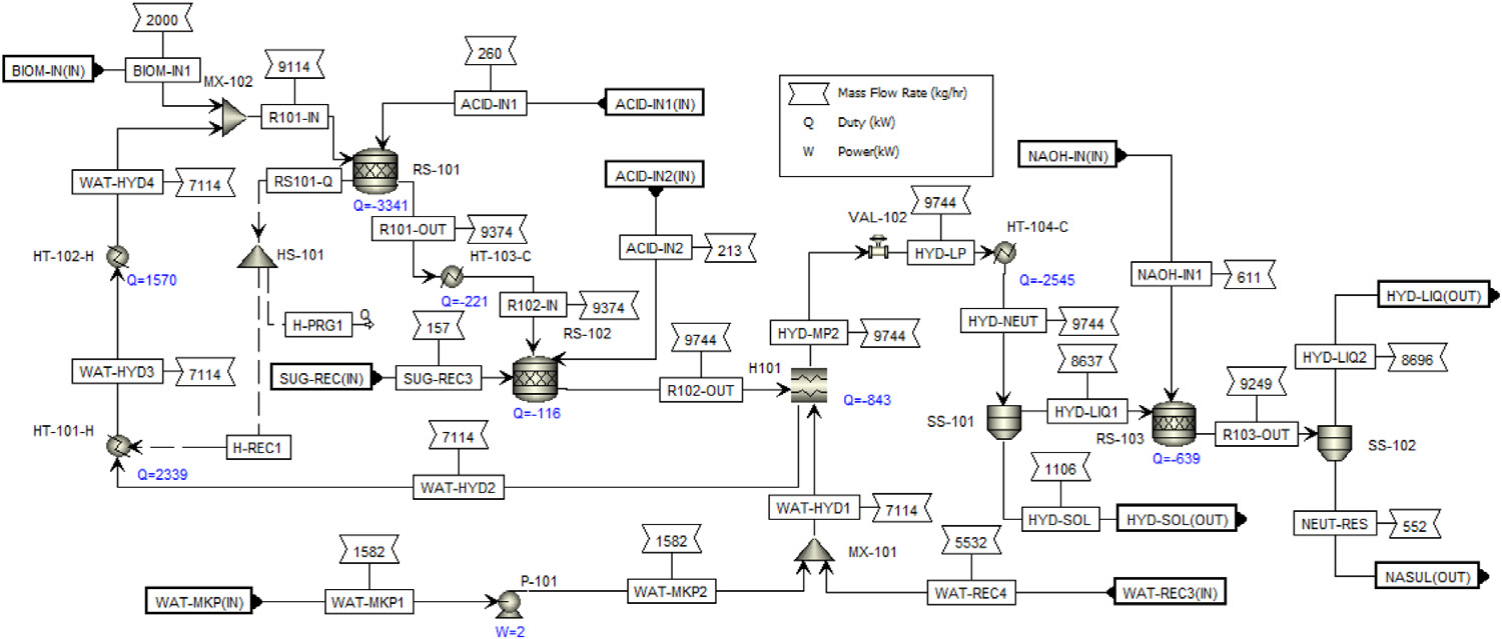
ACID-HYD Hierarchy block details for EL production from RS. This flowsheet includes the two step acid hydrolysis of RS, acid neutralization and solids separation from liquid hydrolyzed.; M: MHeatX; MX: mixer; HS: heat splitter; HT: heater; P: pump; RS: RStoic; SS: solid separator; VAL: valve.

**Fig. 3:**
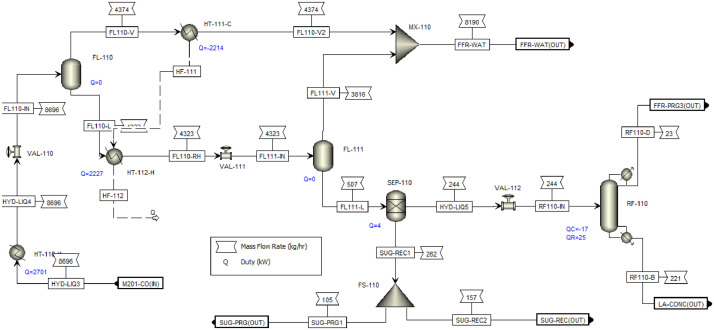
HYD-SEP Hierarchy block details for EL production from RS. This flowsheet includes a two-step flash evaporation for remove water and FFR from the hydrolyzed stream, sugar recovery and a distillation for LA purification. FL: flash; FS: flow splitter; MX: mixer; HT: heater; P: pump; RF: RadFrac; SEP: sep-2; VAL: valve.

**Fig. 4:**
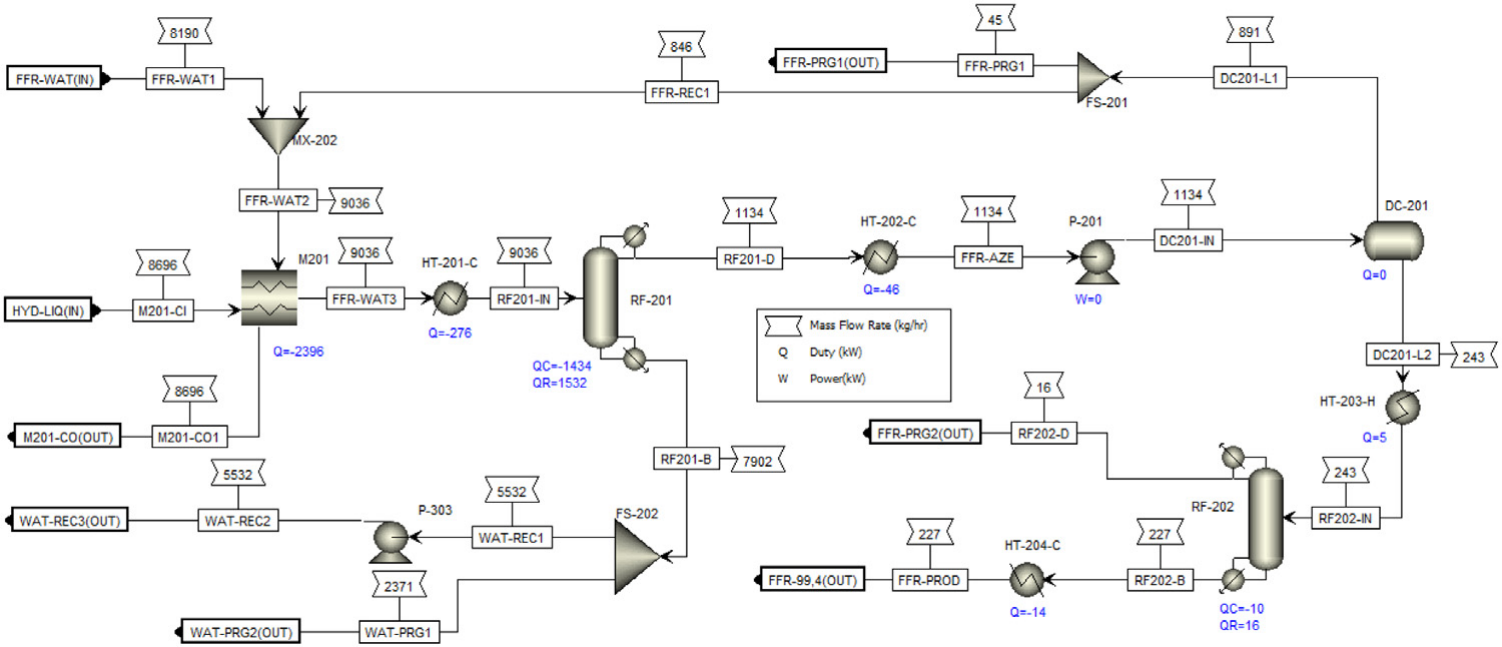
FFR-SEP Hierarchy block details for EL production from RS. This flowsheet includes the two step distillation of FFR with a decanter unit. DC: decanter; FS: flow splitter; M: MHeatX; MX: mixer; HT: heater; P: pump; RF: RadFrac.

**Fig. 5:**
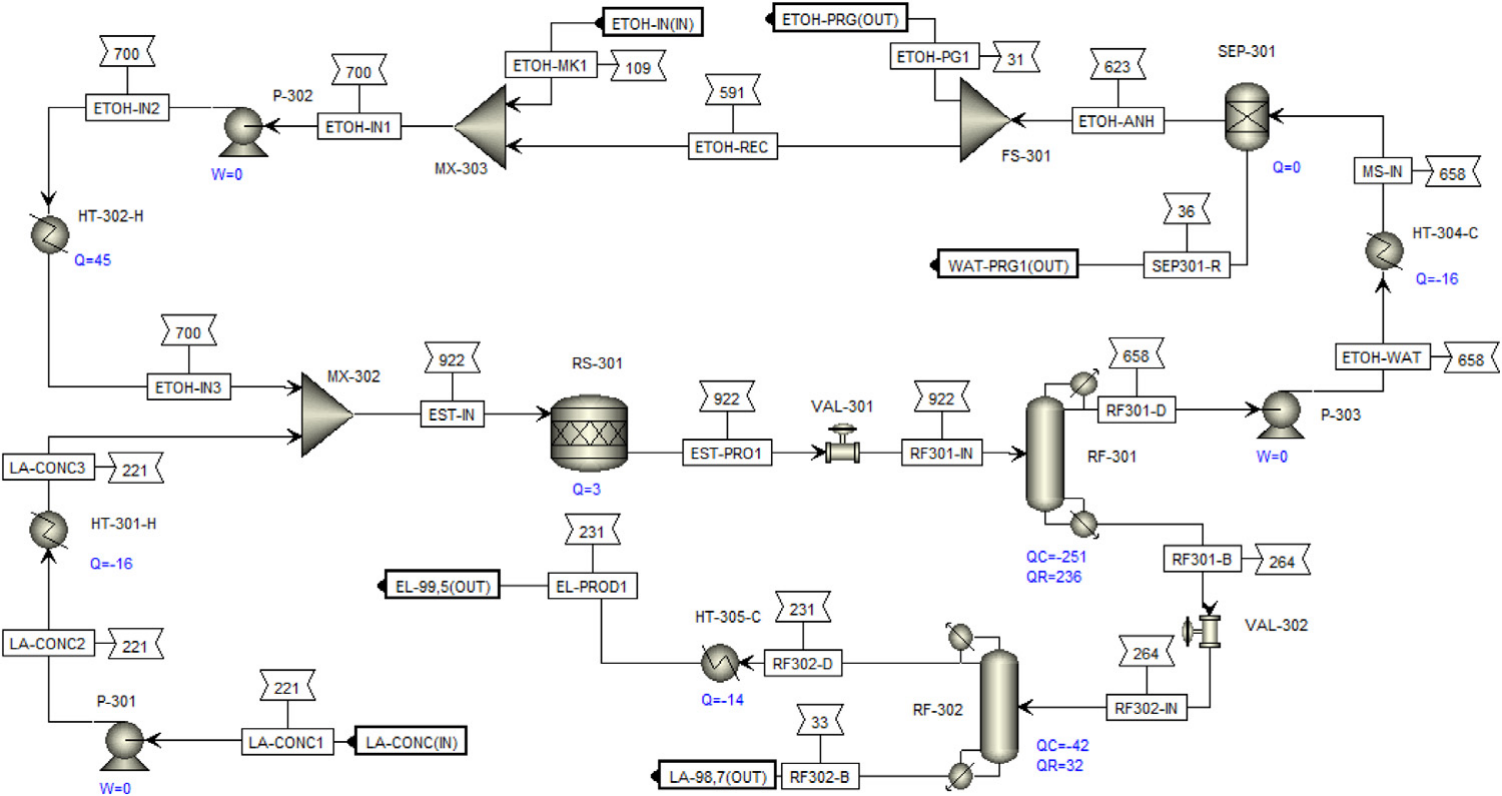
ESTERIF Hierarchy block details for EL production from RS. This flowsheet includes the esterification reaction, two step distillation for EL and LA purification, and ethanol recovery. FS: flow splitter; MX: mixer; HT: heater; P: pump; RF: RadFrac; RS: RStoic; SEP: sep-2; VAL: valve.

**Fig. 6:**
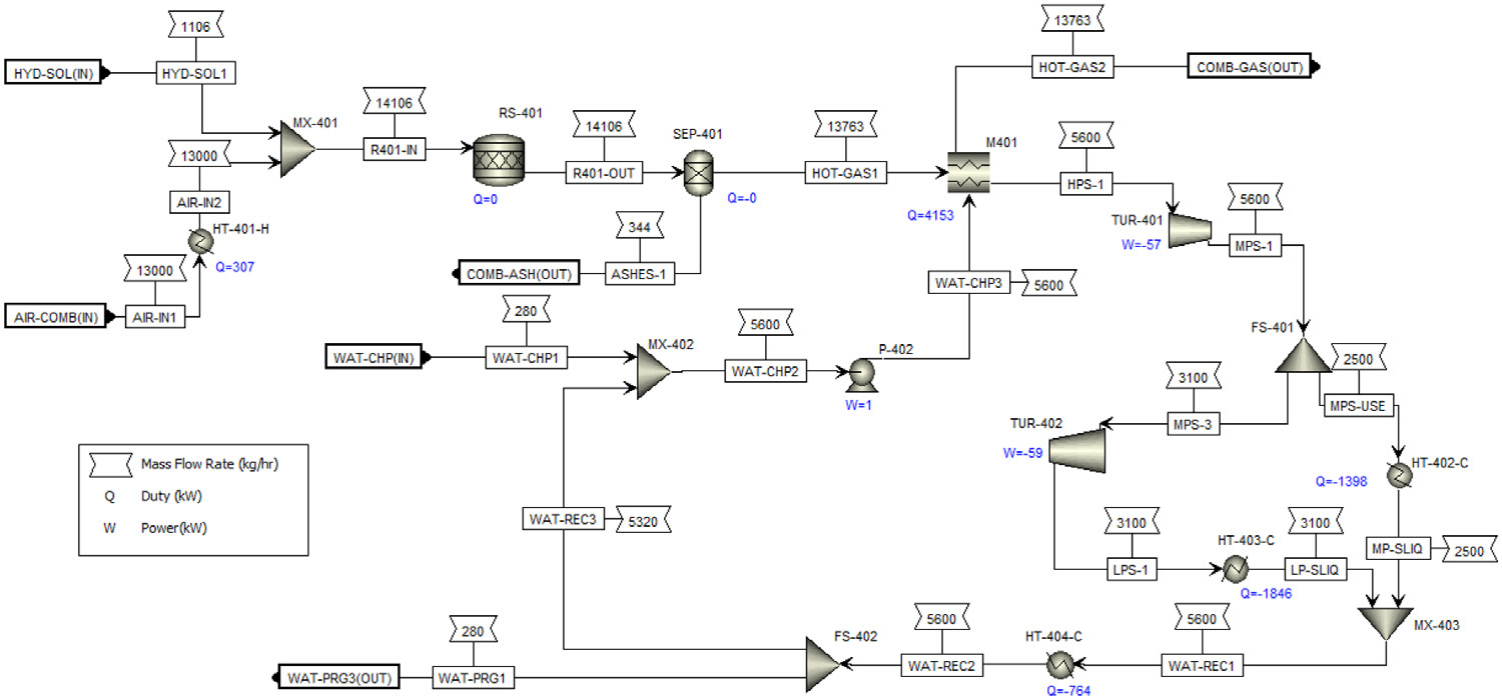
HEAT-GEN Hierarchy block details for EL production from RS including Heat Generation. This flowsheet includes the combustion of solid hydrolyzed and a Rankine cycle for LPS and MPS generation. FS: flow splitter; HT: heater; M: MHeatX; MX: mixer; P: pump; S; RF: RadFrac; SEP: sep-2; TUR: turbine; VAL: valve.

**Fig. 7:**
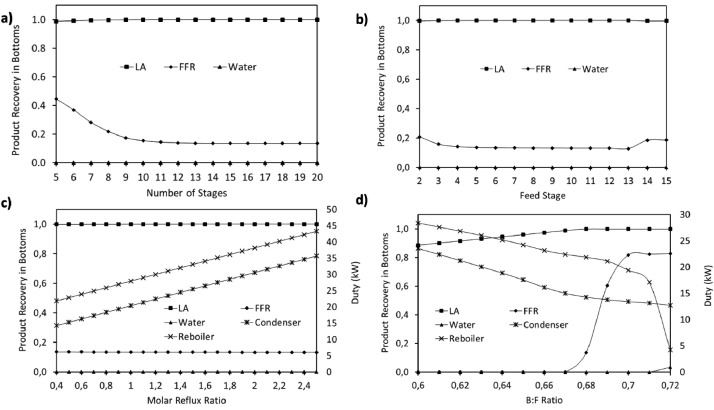
Sensitivity analysis for RF-110. a) Number of stages; b) Feed stage; c) Molar reflux ratio; d) Bottoms to feed ratio. LA = levulinic acid; FFR = furfural.

**Fig. 8:**
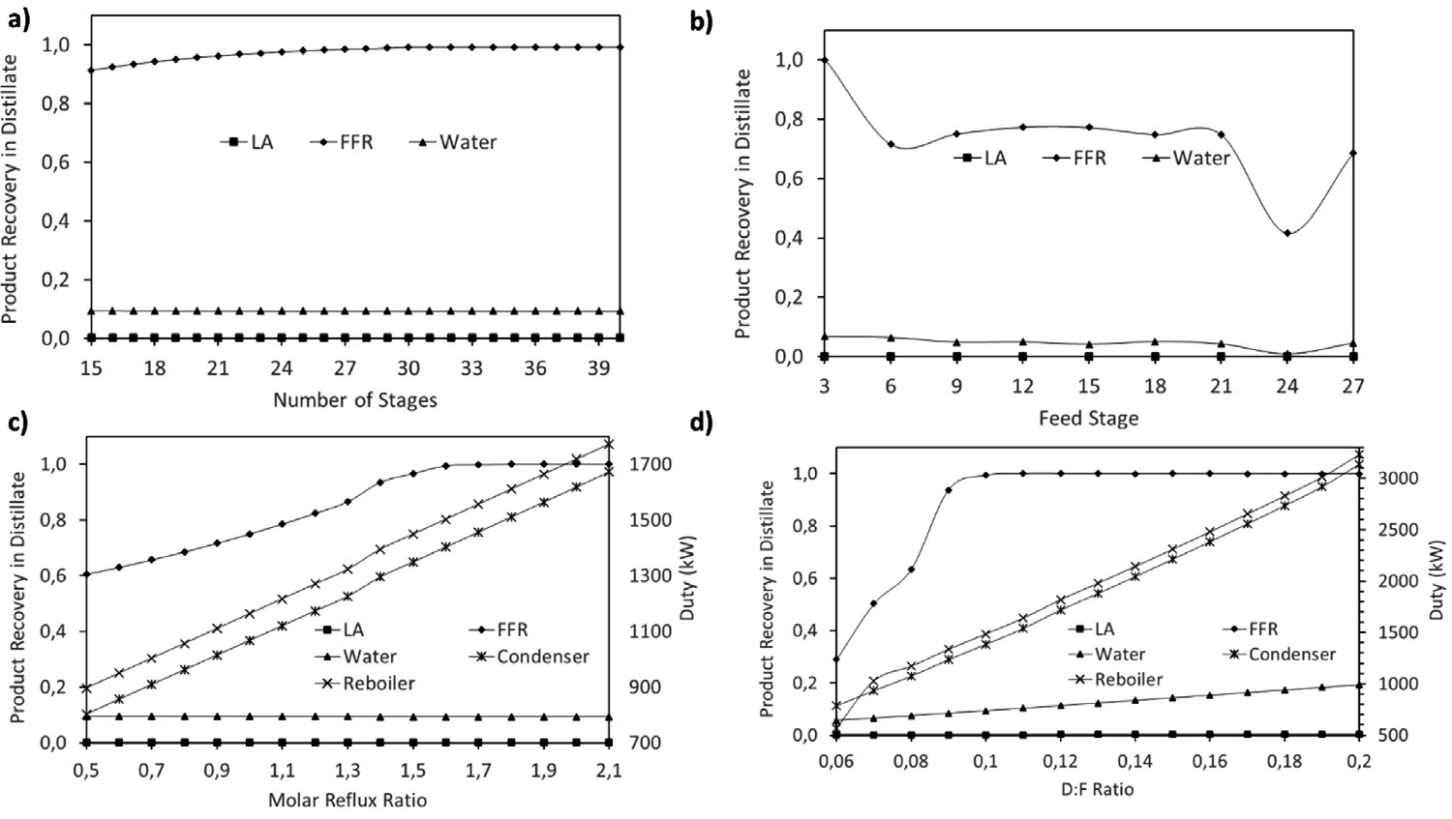
Sensitivity analysis for RF-201. a) Number of stages; b) Feed stage; c) Molar reflux ratio; d) Distillate to feed ratio. LA = levulinic acid; FFR = furfural.

**Fig. 9:**
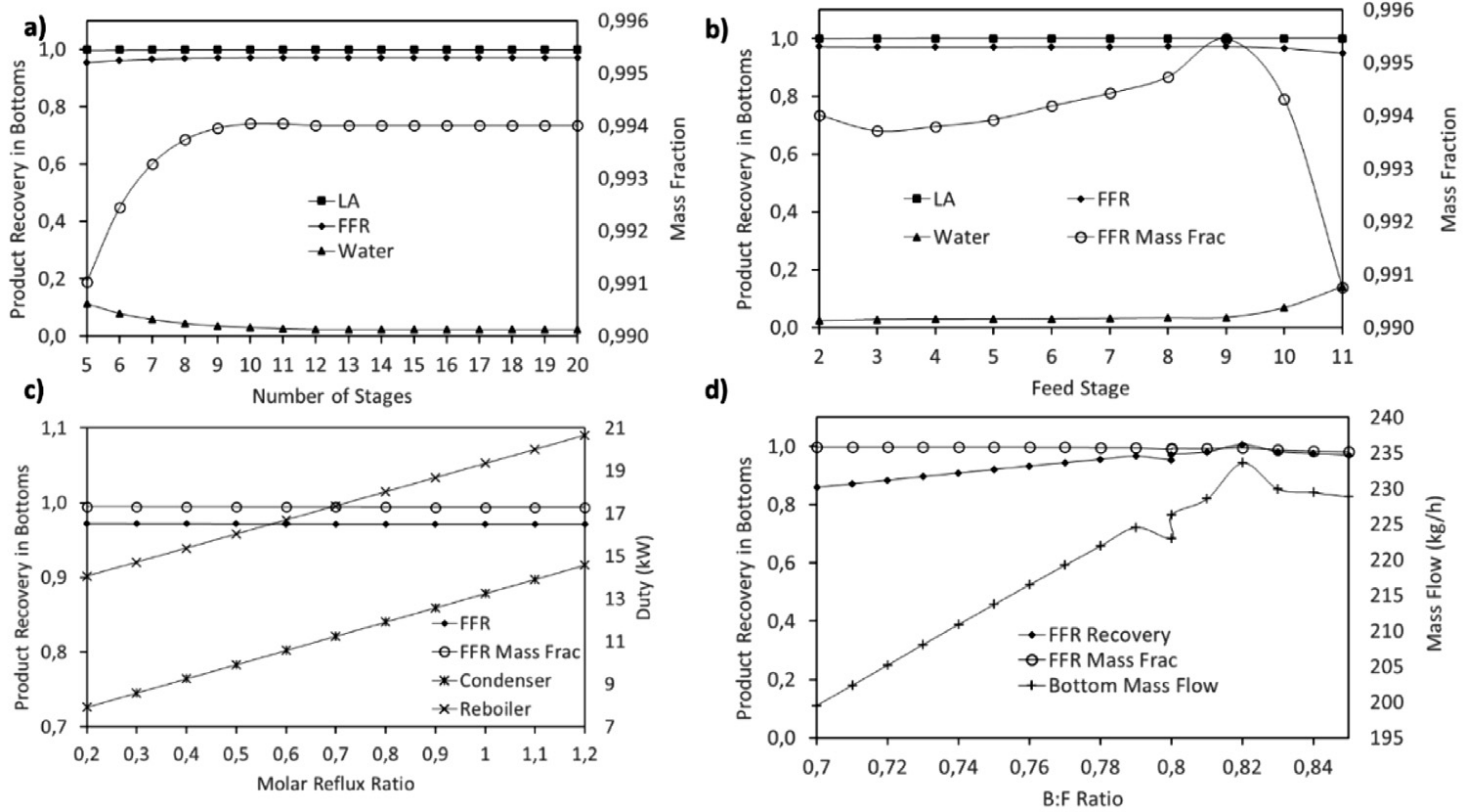
Sensitivity analysis for RF-202. a) Number of stages; b) Feed stage; c) Molar reflux ratio; d) Bottoms to feed ratio. LA = levulinic acid; FFR = furfural.

**Fig. 10:**
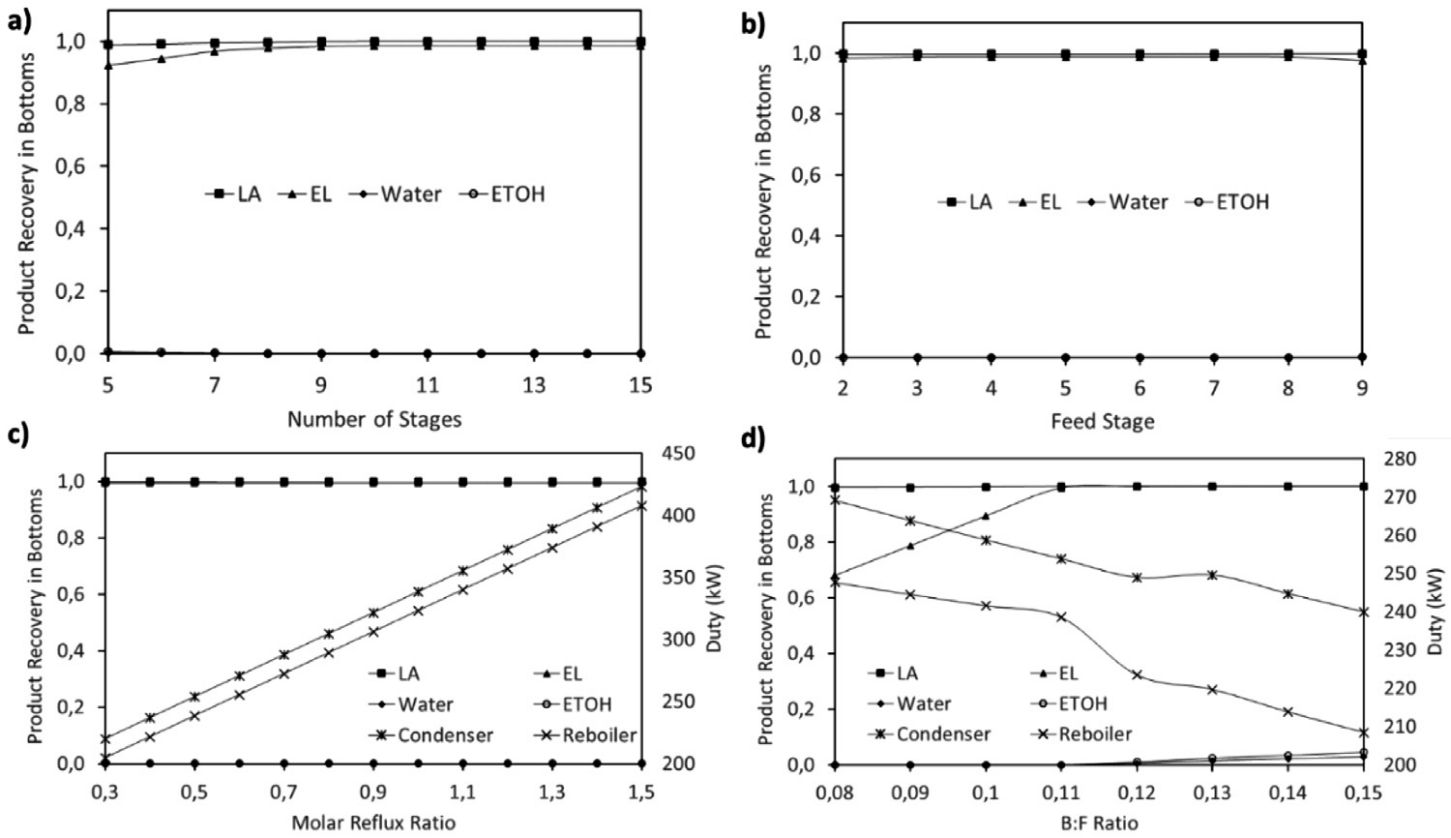
Sensitivity analysis for RF-301. a) Number of stages; b) Feed stage; c) Molar reflux ratio; d) Bottoms to feed ratio. LA = levulinic acid; EL = ethyl levulinate; ETOH = ethanol.

**Fig. 11:**
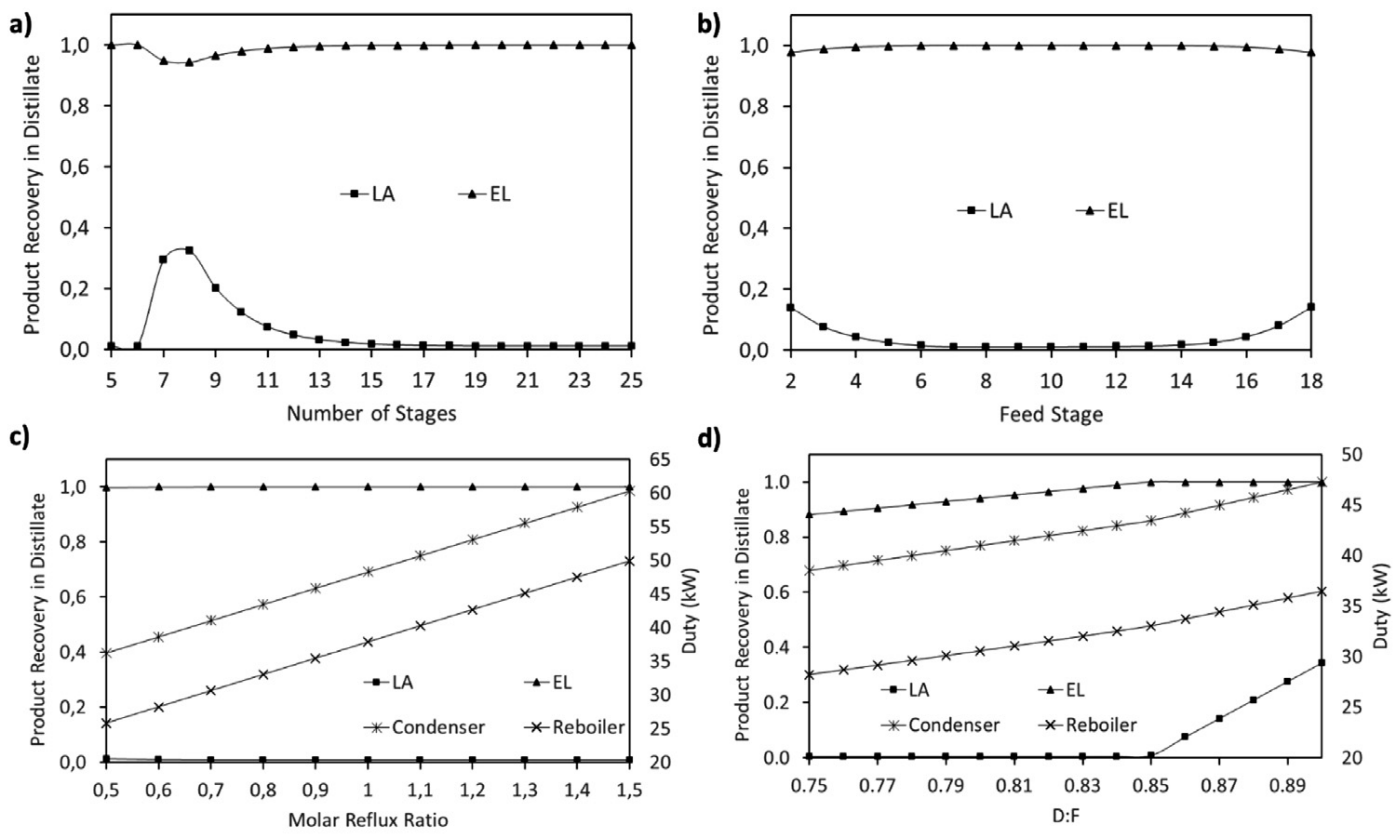
Sensitivity analysis for RF-302. a) Number of stages; b) Feed stage; c) Molar reflux ratio; d) Distillate to feed ratio. LA = levulinic acid; EL = ethyl levulinate.

This article shows the Life Cycle Inventory (LCI) of the foreground system needed to perform a Life Cycle Assessment (LCA) of the production of ethyl levulinate (EL) from Colombian rice straw (RS). These data give transparency to the main results shown in the reference article [1]. LCI was gathered from process simulation using Aspen Plus v.12 (AspenTech, Bedford, USA), Ecoinvent database v.3.4, scientific, academic reports, and websites. Table 1 shows the data collection sources for the LCI construction. Table 2 shows the proximate analysis of RS coming from the Orinoquia Region (Colombia). Table 3 depicts the LCI of the paddy rice cultivation stage. Fig. 1 presents the main flowsheet associated with the conversion of RS into EL. Herein, five hierarchy blocks were employed. Fig. 2 shows the flowsheet for the ACID-HYD block that represents the hydrolysis of RS. Fig. 3 shows the detailed purification (HYD-SEP, in Fig. 1) of LA and furfural (FFR). Fig. 4 shows the detailed purification of FFR (FFR-SEP, in Fig. 1). Fig. 5 depicts the ESTERIF block that simulated the esterification of LA with ethanol to produce EL and its subsequent purification through a separation train. Lastly, Fig. 6 portrays the combustion of solid waste to produce low pressure steam (LPS) and medium pressure steam (MPS). Detailed information of subroutines for the aforecited process is described in Tables 4 and 5. Table 6 presents the list of all reactions used for the *RStoics* units. Table 7 shows the operating conditions for the five distillation units. Sensitivity analysis of these distillation columns is briefly shown in Fig. 7 (RF-110), Fig. 8 (RF-201), Fig. 9 (RF-202), Fig. 10 (RF-301), and Fig. 11 (RF-302).

**Table 1:** Data collection for the construction of the LCI in each stage of the product system.

Process stage	Methodology
Biomass cultivation	Ecoinvent Literature review
Biomass harvesting	
Acid Hydrolysis	Ecoinvent Literature review Process simulation in Aspen Plus
Solid Combustion	
LA purification	
EL production

**Table 2:** Proximate analysis of rice and carbon content on each fraction.

Analysis	Unit	Value	g C/kg
Crude protein	% Dry matter	14.2	530
Crude Fiber	% Dry matter	4.10	440
NDF	% Dry matter	12.4	440
ADF	% Dry matter	3.2	440
Lignin	% Dry matter	1.2	645
Lipids	% Dry matter	13.2	750
Starch	% Dry matter	42.0	440
Total sugars	% Dry matter	3.8	440

NDF: Neutral detergent fiber; ADF: acid detergent fiber.

**Table 3:** Life Cycle Inventory for rice culture in Orinoquia region in Colombia.

Component	Stream type	Unit	Amount
Paddy rice	Output	kg	1000.000
Rice Straw	Output	kg	1400.000
Land	Input	ha	0.201
Ammonia	Input	kg	104.340
P_2_O_5_	Input	kg	7.243
K_2_O	Input	kg	31.590
Diesel	Input	L	25.1
Irrigation	Input	m^3^	1047.561
Water (rain)	Input	m^3^	2879.276
CO_2_ (capture)	Input	kg	3687.113
Hydrocarbons	Emission to air	kg	0.032
Nitrogen oxides	Emission to air	kg	1.733
CO	Emission to air	kg	0.317
CO_2_	Emission to air	kg	74.761
Particulate Matter	Emission to air	kg	0.040
SO_x_	Emission to air	kg	0.023
NH_3_	Emission to air	kg	0.806
NO_x_	Emission to air	kg	0.872
N_2_O	Emission to air	kg	0.461
CH_4_	Emission to air	kg	16.711
Nitrates	Emission to water	kg	36.937
Phosphate to surface water	Emission to water	kg	0.055

**Table 4:** Description of main subroutines to produce ethyl levulinate from rice straw.

Hierarchy block	Aspen subroutine	Description	Conditions	Assumptions
ACID-HYD	RS-101	Reactor for the first stage of dilute acid hydrolysis	T: 210°C P: 20 bar Reactions: reactions 1 to 5 in Table 6 Utility: cooling air Q: -12,029.3 MJ/h	Stoichiometric reactions. No reversible reactions
ACID-HYD	RS-102	Reactor for the second stage of dilute acid hydrolysis	T: 190°C P: 18 bar Reactions: reactions 6 to 8 in Table 6 Utility: cooling air Q: -418.7 MJ/h	Stoichiometric reactions. No reversible reactions
ACID-HYD	RS-103	Reactor for sulfuric acid neutralization with sodium hydroxide	T: 70°C P: 6 bar Reactions: reaction 17 in Table 6 Utility: cooling air Q: -2,300.1 MJ/h	Stoichiometric reactions. No reversible reactions
ACID-HYD	H101	Heat exchanger for preheating of hydrolysis water	Out temperature of cold stream 185°C Temperature difference: 10°C	Geometry and rigorous design avoided
ACID-HYD	SS-101	Solid separator for solid hydrolyzed separation from liquid hydrolyzed stream	T: 70°C P: 6 bar Fraction of solids to solid outlet: 0.999 Fraction of liquid to liquid outlet: 0.99	No particle size distribution
ACID-HYD	SS-102	Solid separator for sodium sulfate separation from liquid hydrolyzed stream	T: 70°C P: 6 bar Fraction of solids to solid outlet: 0.9999 Fraction of liquid to liquid outlet: 0.999	No particle size distribution
ACID-HYD	P-101	Pump for water make up for dilute acid hydrolysis	P out: 20 bar Efficiency: 70% Utility: electricity	Only liquid phase
ACID-HYD	HT-101-H	Heat exchanger for hydrolysis water preheating	P: 20 bar Utility: HPS Outlet temperature: 212.8°C Outlet vapor fraction: 0.57 Q: 8420.1 MJ/h	No pressure drop considered
ACID-HYD	HT-102-H	Heat exchanger for hydrolysis water evaporation	P: 20 bar Utility: HPS Outlet temperature: 215°C Outlet vapor fraction: 0.999 Q: 5,651.2 MJ/h	No pressure drop considered
ACID-HYD	HT-103-H	Heat exchanger for cooling hydrolysate stream from RS-101 to RS-102	Outlet temperature: 190°C P: 18 bar Utility: Cooling air Q: -797.3 MJ/h	No pressure drop considered
ACID-HYD	VAL-102	Valve for pressure reduction of hydrolysate stream	Outlet pressure: 6 bar Valid phases: liquid and vapor	Adiabatic
ACID-HYD	HT-104-C	Heat exchanger for hydrolysate stream cooling previous to neutralization	Outlet temperature: 70°C P: 6 bar Utility: Cooling air Q: -9,163.5 MJ/h	No pressure drop considered
HYD-SEP	FL-110	First flash unit for LA and FFR separation from liquid hydrolyzed stream	Pressure drop: 0 bar	Adiabatic
HYD-SEP	FL-111	Second flash unit for LA and FFR separation from liquid hydrolyzed stream	Pressure drop: 0 bar	Adiabatic
HYD-SEP	SEP-110	Separation unit for unreacted sugar recovery	Split fraction for fructose: 0.9999 Split fraction for xylose: 0.9999 Split fraction for water: 0.2 P: 1.2 bar	Non-rigorous unit
HYD-SEP	RF-110	Distillation column for levulinic acid purification	NS: 14 FS: 6 (above) Molar reflux ratio: 0.7 Bottoms to feed ratio: 0.68 Convergence: strongly non-ideal liquid Condenser pressure: 0.2 bar Column pressure dop: 0.25 bar Murphree efficiency: 65% Condenser utility: cooling water Condenser Q: -62.4 MJ/h Reboiler utility: HPS Reboiler Q: 89.6 MJ/h	Internal design not considered
HYD-SEP	VAL-110	Valve for pressure drop required to FL-110 unit	Outlet pressure: 1.75 bar	Adiabatic
HYD-SEP	VAL-111	Valve for pressure drop required to FL-111 unit	Outlet pressure: 1.7 bar	Adiabatic
HYD-SEP	VAL-112	Valve for pressure drop required to RF-110 unit	Outlet pressure: 0.3 bar	Adiabatic
HYD-SEP	HT-110-H	Heat exchanger for heating requirement for FL-110 unit	Outlet temperature: 134°C P: 3 bar Utility: MPS Q: 9,723.6 MJ/h	No pressure drop considered
HYD-SEP	HT-111-C	Heat exchanger for cooling vapor stream from FL-110 unit	Outlet vapor fraction: 0.12 Pressure drop: 0 bar Q: -7971.6 MJ/h	No utility required
HYD-SEP	HT-112-H	Heat exchanger for heating requirement for FL-111 unit	Outlet temperature: 125°C Pressure drop: 0 bar Q: 46.19 MJ/h	No utility required
FFR-SEP	M201	Heat exchanger for heat integration of liquid stream feed to furfural azeotropic distillation	Cold stream outlet temperature: 102°C	No pressure drop considered
FFR-SEP	RF-201	Distillation column for azeotropic distillation of furfural from water	NS: 30 FS: 2 (above) Molar reflux ratio: 1.7 Distillate to feed ratio: 0.1 Convergence: azeotropic Condenser pressure: 1 bar Column pressure dop: 0.4 bar Murphree efficiency: 65% Condenser utility: cooling water Condenser Q: -5,160.6 MJ/h Reboiler utility: LPS Reboiler Q: 5,513.3 MJ/h	Internal design not considered
FFR-SEP	RF-202	Distillation column for furfural purification	NS: 12 FS: 6 (above) Molar reflux ratio: 0.5 Bottoms to feed ratio: 0.8 Convergence: standard Condenser pressure: 1 bar Column pressure dop: 0.3bar Murphree efficiency: 65% Condenser utility: cooling water Condenser Q: -35.8 MJ/h Reboiler utility: HPS Reboiler Q: 57.8 MJ/h	Internal design not considered
FFR-SEP	HT-201-C	Heat exchanger for cooling of liquid stream feed to furfural azeotropic distillation	Outlet temperature: 99°C Outlet pressure: 1.02 bar Utility: cooling air Q: -994.5 MJ/h	No pressure drop considered
FFR-SEP	HT-202-C	Heat exchanger for cooling of distillate stream from RF-201 unit to DC-201 unit	Outlet temperature: 60°C Outlet pressure: 1 bar Utility: cooling water Q: -164.5 MJ/h	No pressure drop considered
FFR-SEP	HT-203-H	Heat exchanger for heating requirement for RF-202 unit	Outlet temperature: 96°C Outlet pressure: 1.16 bar Utility: LPS Q: 16.4 MJ/h	No pressure drop considered
FFR-SEP	HT-204-C	Heat exchanger for cooling of furfural product stream	Outlet temperature: 38°C Outlet pressure: 1 bar Utility: cooling air Q: -50.5 MJ/h	No pressure drop considered
FFR-SEP	P-201	Pump for pressure adjust required for DC-201 unit	P out: 1.3 bar Efficiency: 70% Utility: electricity	Only liquid phase
FFR-SEP	P-303	Pump for water recycle for dilute acid hydrolysis	P out: 20 bar Efficiency: 70% Utility: electricity	Only liquid phase
FFR-SEP	DC-201	Decanter unit for furfural rich phase separation from distillate stream from RF-201	T: 60°C P: 1.3 bar Key components for separation: water and furfural	-
ESTERIF	RS-301	Esterification reactor	T: 120°C P: 4.5 bar Reactions: 9 reaction in Table 6 Utility: MPS Q: 9.1 MJ/h	Stoichiometric reactions. No reversible reactions
ESTERIF	RF-301	Distillation column for water and ethanol separation from esterification reaction outlet stream	NS: 10 FS: 6 (above) Molar reflux ratio: 0.5 Bottoms to feed ratio: 0.11 Convergence: Strongly non-ideal liquid Condenser pressure: 1 bar Column pressure dop: 0.3 bar Murphree efficiency: 65% Condenser utility: cooling water Condenser Q: -905.1 MJ/h Reboiler utility: HPS Reboiler Q: 850.8 MJ/h	Internal design not considered
ESTERIF	RF-302	Distillation column for ethyl levulinate purification	NS: 19 FS: 8 (above) Molar reflux ratio: 0.8 Distillate to feed ratio: 0.85 Convergence: Strongly non-ideal liquid Condenser pressure: 0.2 bar Column pressure dop: 0.25 bar Murphree efficiency: 65% Condenser utility: cooling water Condenser Q: -149.9 MJ/h Reboiler utility: HPS Reboiler Q: 115.1 MJ/h	Internal design not considered
ESTERIF	SEP-301	Molecular sieve separation for ethanol recycling	Split fraction for water: 0.0001 Split fraction for ethanol: 0.996 Split fraction for levulinic acid: 0.0001 Split fraction for ethyl levulinate: 0.0001 P: 1.5 bar	Non-rigorous unit
ESTERIF	VAL-301	Valve for pressure drop required to RF-301 unit	Outlet pressure: 1.16 bar	Adiabatic
ESTERIF	VAL-302	Valve for pressure drop required to RF-302 unit	Outlet pressure: 0.4 bar	Adiabatic
ESTERIF	P-301	Pump for levulinic acid stream pressure adjust required for esterification reaction	P out: 4.5 bar Efficiency: 70% Utility: electricity	Only liquid phase
ESTERIF	P-302	Pump for ethanol stream pressure adjust required for esterification reaction	P out: 4.5 bar Efficiency: 70% Utility: electricity	Only liquid phase
ESTERIF	P-303	Pump for pressure adjust of distillate stream from RF-301 to SEP-301	P out: 1.5 bar Efficiency: 70% Utility: electricity	Only liquid phase
ESTERIF	HT-301-H	Heat exchanger for levulinic acid stream cooling required for esterification reaction	Outlet temperature: 120°C Outlet pressure: 4.5 bar Utility: cooling air Q: -57.2 MJ/h	No pressure drop considered
ESTERIF	HT-302-H	Heat exchanger for ethanol stream heating required for esterification reaction	Outlet temperature: 120°C Outlet pressure: 4.5 bar Utility: MPS Q: 163.3 MJ/h	No pressure drop considered
ESTERIF	HT-304-C	Heat exchanger for cooling of distillate stream from RF-301 to SEP-301	Outlet temperature: 50°C Outlet pressure: 1.5 bar Utility: cooling air Q: -55.9 MJ/h	No pressure drop considered
ESTERIF	HT-305-C	Heat exchanger for cooling of ethyl levulinate product stream	Outlet temperature: 28°C Outlet pressure: 1 bar Utility: cooling water Q: -51.7 MJ/h	No pressure drop considered
HEAT-GEN	RS-401	Reactor for solid hydrolyzed stream combustion	T: 904°C P: 10 bar Reactions: 10 to 16 reactions in Table 6	Stoichiometric reactions. No reversible reactions. Complete combustion Adiabatic reactor. Excess air to temperature control.
HEAT-GEN	SEP-401	Separation of ashes from combustion reactor	Split fraction for solid components: 0.0001 Split fraction for other components: 0.9999	No pressure drop considered
HEAT-GEN	M401	Heat exchanger that simulates furnace of steam generation cycle	Hot stream outlet temperature: 40°C Hot stream outlet pressure: 10 bar Cold stream outlet temperature: 5 bar	No pressure drop considered
HEAT-GEN	HT-401-H	Heat exchanger for combustion air preheating	Outlet temperature: 110°C Outlet pressure: 10 bar Utility: MPS Q: 1106.6 MJ/h	No pressure drop considered
HEAT-GEN	HT-402-C	Heat exchanger that simulates the condensing of generated MPS used in the overall process	Outlet temperature: 174°C Outlet vapor fraction: 0 Utility: none Q: -5,181.2	No pressure drop considered
HEAT-GEN	HT-403-C	Heat exchanger that simulates the condensing of generated LPS used in the overall process	Outlet temperature: 124°C Outlet vapor pressure: 0 Utility: none Q: -6,810.1 MJ/h	No pressure drop considered
HEAT-GEN	HT-404-C	Heat exchanger that simulates condenser of steam generation cycle	Outlet temperature: 30°C Outlet vapor fraction: 0 Utility: cooling air Q: -2,750.9 MJ/h	No pressure drop considered
HEAT-GEN	P-402	Pump for water pressurization for steam generation	P out: 5 bar Efficiency: 70% Utility: electricity	Only liquid phase
HEAT-GEN	TUR-401	Turbine for MPS generation	Discharge pressure: 3.7 bar Isentropic efficiency: 80% Mechanical efficiency: 80%	Isentropic using GPSA method
HEAT-GEN	TUR-402	Turbine for LPS generation	Discharge pressure: 1.95 bar Isentropic efficiency: 80% Mechanical efficiency: 80%	Isentropic using GPSA method

T: temperature; P: pressure; FS: feed stage; NS: number of stages; Q: net duty; LPS: low pressure steam; MPS: medium pressure steam; HPS: high pressure steam.

**Table 5:** Subroutines employed to simulate the production of ethyl levulinate from rice Straw.

Hierarchy block	Subroutine	Purpose
ACID-HYD	RS-101	Reactor for the first stage of dilute acid hydrolysis using sulfuric acid
ACID-HYD	RS-102	Reactor for the second stage of dilute acid hydrolysis using sulfuric aid
ACID-HYD	RS-103	Reactor for sulfuric acid neutralization with sodium hydroxide
ACID-HYD	H101	Heat exchanger for preheating hydrolysis water using heat integration of hydrolysed outlet stream (liquid and solid) and the hydrolysis water (includes make-up water and recycled water from FFR distillation)
ACID-HYD	SS-101	Solid separator for solid hydrolyzed separation from hydrolyzed outlet stream
ACID-HYD	SS-102	Solid separator for sodium sulfate separation from liquid hydrolyzed stream from SS-101
ACID-HYD	P-101	Pump for water make up for dilute acid hydrolysis
ACID-HYD	HT-101-H	Heat exchanger for hydrolysis water preheating
ACID-HYD	HT-102-H	Heat exchanger for hydrolysis water evaporation
ACID-HYD	HT-103-H	Heat exchanger for cooling hydrolysate stream from RS-101 to RS-102
ACID-HYD	VAL-102	Valve for pressure reduction of hydrolysate stream
ACID-HYD	HT-104-C	Heat exchanger for hydrolysate stream cooling previous to neutralization
HYD-SEP	FL-110	First flash unit for LA and FFR separation from liquid hydrolyzed stream
HYD-SEP	FL-111	Second flash unit for LA and FFR separation from liquid hydrolyzed stream
HYD-SEP	SEP-110	Separation unit for unreacted sugars recovery
HYD-SEP	RF-110	Distillation column for levulinic acid purification
HYD-SEP	VAL-110	Valve for pressure drop required to FL-110 unit
HYD-SEP	VAL-111	Valve for pressure drop required to FL-111 unit
HYD-SEP	VAL-112	Valve for pressure drop required to RF-110 unit
HYD-SEP	HT-110-H	Heat exchanger for heating requirement for FL-110 unit
HYD-SEP	HT-111-C	Heat exchanger for cooling vapor stream from FL-110 unit
HYD-SEP	HT-112-H	Heat exchanger for heating requirement for FL-111 unit
FFR-SEP	M201	Heat exchanger for heat integration of liquid stream feed to furfural azeotropic distillation
FFR-SEP	RF-201	Distillation column for azeotropic distillation of furfural from water
FFR-SEP	RF-202	Distillation column for furfural purification
FFR-SEP	HT-201-C	Heat exchanger for cooling of liquid stream feed to furfural azeotropic distillation
FFR-SEP	HT-202-C	Heat exchanger for cooling of distillate stream from RF-201 unit to DC-201 unit
FFR-SEP	HT-203-H	Heat exchanger for heating requirement for RF-202 unit
FFR-SEP	HT-204-C	Heat exchanger for cooling of furfural product stream
FFR-SEP	P-201	Pump for pressure adjust required for DC-201 unit
FFR-SEP	P-303	Pump for water recycle for dilute acid hydrolysis
FFR-SEP	DC-201	Decanter unit for furfural rich phase separation from distillate stream from RF-201
ESTERIF	RS-301	Esterification reactor using ethanol as solvent and as esterification reactant
ESTERIF	RF-301	Distillation column for water and ethanol separation from esterification reaction outlet stream
ESTERIF	RF-302	Distillation column for EL and LA purification
ESTERIF	SEP-301	Molecular sieve separation for ethanol recycling
ESTERIF	VAL-301	Valve for pressure drop required to RF-301 unit
ESTERIF	VAL-302	Valve for pressure drop required to RF-302 unit
ESTERIF	P-301	Pump for levulinic acid stream pressure adjust required for esterification reaction
ESTERIF	P-302	Pump for ethanol stream pressure adjust required for esterification reaction
ESTERIF	P-303	Pump for pressure adjust of distillate stream from RF-301 to SEP-301
ESTERIF	HT-301-H	Heat exchanger for levulinic acid stream cooling required for esterification reaction
ESTERIF	HT-302-H	Heat exchanger for ethanol stream heating required for esterification reaction
ESTERIF	HT-304-C	Heat exchanger for cooling of distillate stream from RF-301 to SEP-301
ESTERIF	HT-305-C	Heat exchanger for cooling of ethyl levulinate product stream
HEAT-GEN	RS-401	Reactor for solid hydrolyzed stream combustion, assuming complete combustion and excess air to temperature control
HEAT-GEN	SEP-401	Separation of ashes from combustion reactor
HEAT-GEN	M401	Heat exchanger that simulates furnace of steam generation cycle
HEAT-GEN	HT-401-H	Heat exchanger for combustion air preheating
HEAT-GEN	HT-402-C	Heat exchanger that simulates the condensing of generated MPS used in the overall process
HEAT-GEN	HT-403-C	Heat exchanger that simulates the condensing of generated LPS used in the overall process
HEAT-GEN	HT-404-C	Heat exchanger that simulates condenser of steam generation cycle
HEAT-GEN	P-402	Pump for water pressurization for steam generation
HEAT-GEN	TUR-401	Turbine for MPS generation
HEAT-GEN	TUR-402	Turbine for LPS generation

**Table 6:** Reactions involved in the EL production from rice straw.

#	Reaction	Fractional conversion	Temperature(°C)	Pressure(Bar)
1	Cellulose + H_2_O → Fructose	0.65	210	20
2	Hemicellulose + H_2_O → Xylose	0.82	210	20
3	Hemicellulose + H_2_O → 2.5 AA	0.05	210	20
4	Fructose → Humins + H_2_O	0.1	210	20
5	Xylose → Humins + H_2_O	0.1	210	20
6	Xylose → Furfural + 3H_2_O	0.80	190	18
7	Fructose → HMF + 3H_2_O	0.70	190	18
8	HMF + 2H_2_O → LA + FA	0.999	190	18
9	LA + Ethanol → EL + H_2_O	0.85	120	4.5
10	Cellulose + 6 O_2_ → 5 H_2_O + 6 CO_2_	0.999	904	10
11	Hemicellulose + 5 O_2_ → 4 H_2_O + 5 CO_2_	0.999	904	10
12	Lignin + 10.125 O_2_ → 6.95 H_2_O + 7.3 CO_2_	0.999	904	10
13	Fructose + 6 O_2_ → 6 H_2_O + 6 CO_2_	0.999	904	10
14	Xylose + 5 O_2_ → 5 H_2_O + 5 CO_2_	0.999	904	10
15	Humins (from cellulose) + 6 O_2_ → 5 H_2_O + 6 CO_2_	0.999	904	10
16	Humins (from hemicellulose) + 5 O_2_ → 4 H_2_O + 5 CO_2_	0.999	904	10
17	H_2_SO_4_ + 2 NaOH → Na_2_SO_4_ + 2 H_2_O	0.999	70	6

AA: Acetic acid; HMF: 5-hydroximethylfurfural; LA: Levulinic acid; FA: Formica cid; EL: Ethyl levulinate.

**Table 7:** Distillation columns operation conditions.

Distillation Column	Process Stage	NS	FS	RR	D:F	B:F	TSP (bar)	PD (bar)
RF-110	LA and FFR purification	14	6	0.7	N/A	0.68	0.2	0.25
RF-201	LA and FFR purification	30	2	1.7	0.1	N/A	1.0	0.40
RF-202	LA and FFR purification	12	6	0.5	N/A	0.8	1.0	0.30
RF-301	EL production	10	6	0.5	N/A	0.11	1.0	0.30
RF-302	EL production	19	8	0.8	0.85	N/A	0.2	0.25

NS: number of stages; FS: feed stage; RR: molar reflux ratio; D:F: distillate to feed ratio; B:F: bottoms to feed ratio; TSP: top stage pressure; PD: pressure drop of the column.

**Fig. 12:**
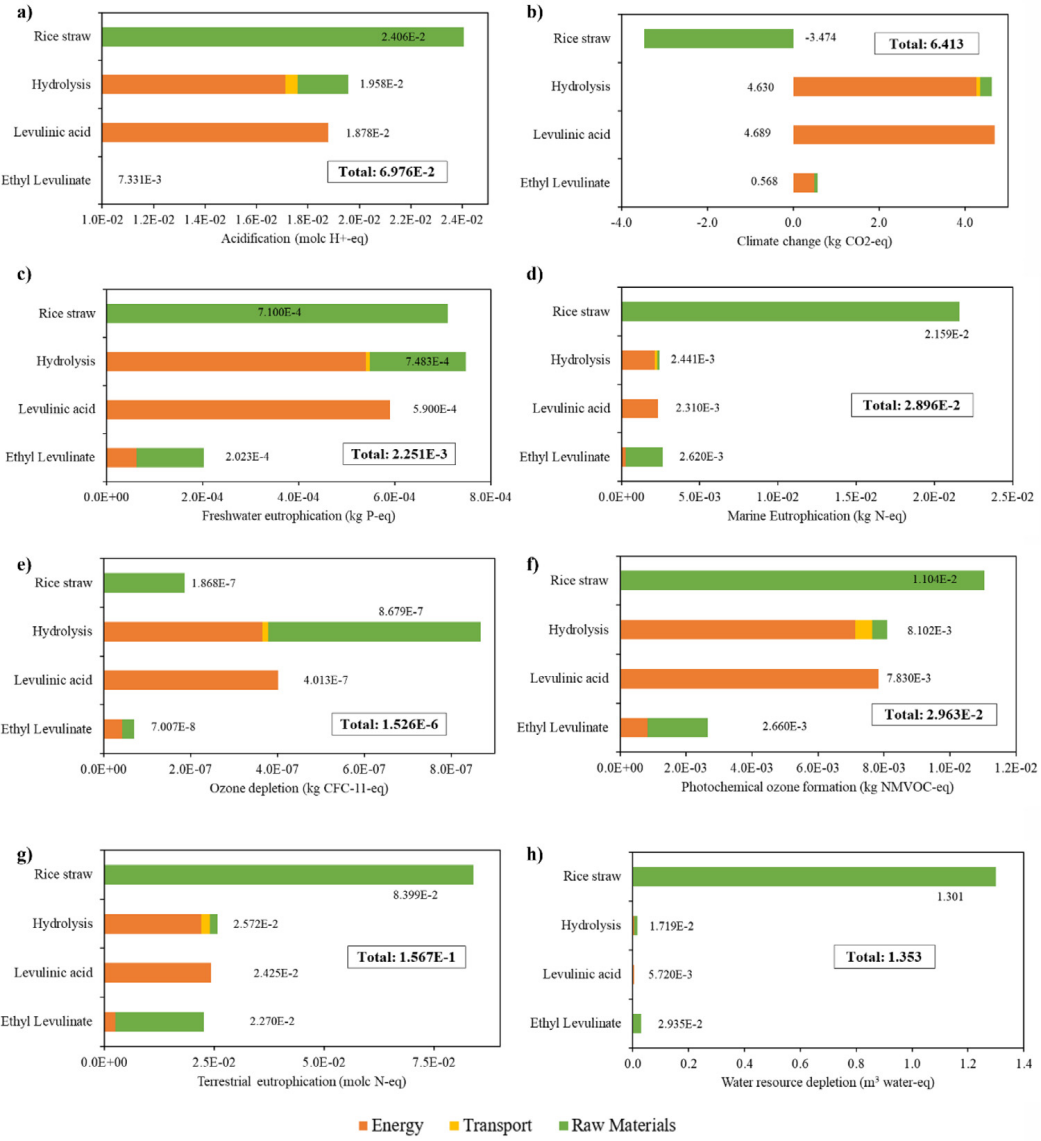
Contribution analysis for base scenario for EL production from RS for a) acidification, b) climate change, c) freshwater eutrophication, d) marine eutrophication, e) ozone depletion, f) photochemical ozone formation, g) terrestrial eutrophication, and h) water resource depletion.

**Fig. 13:**
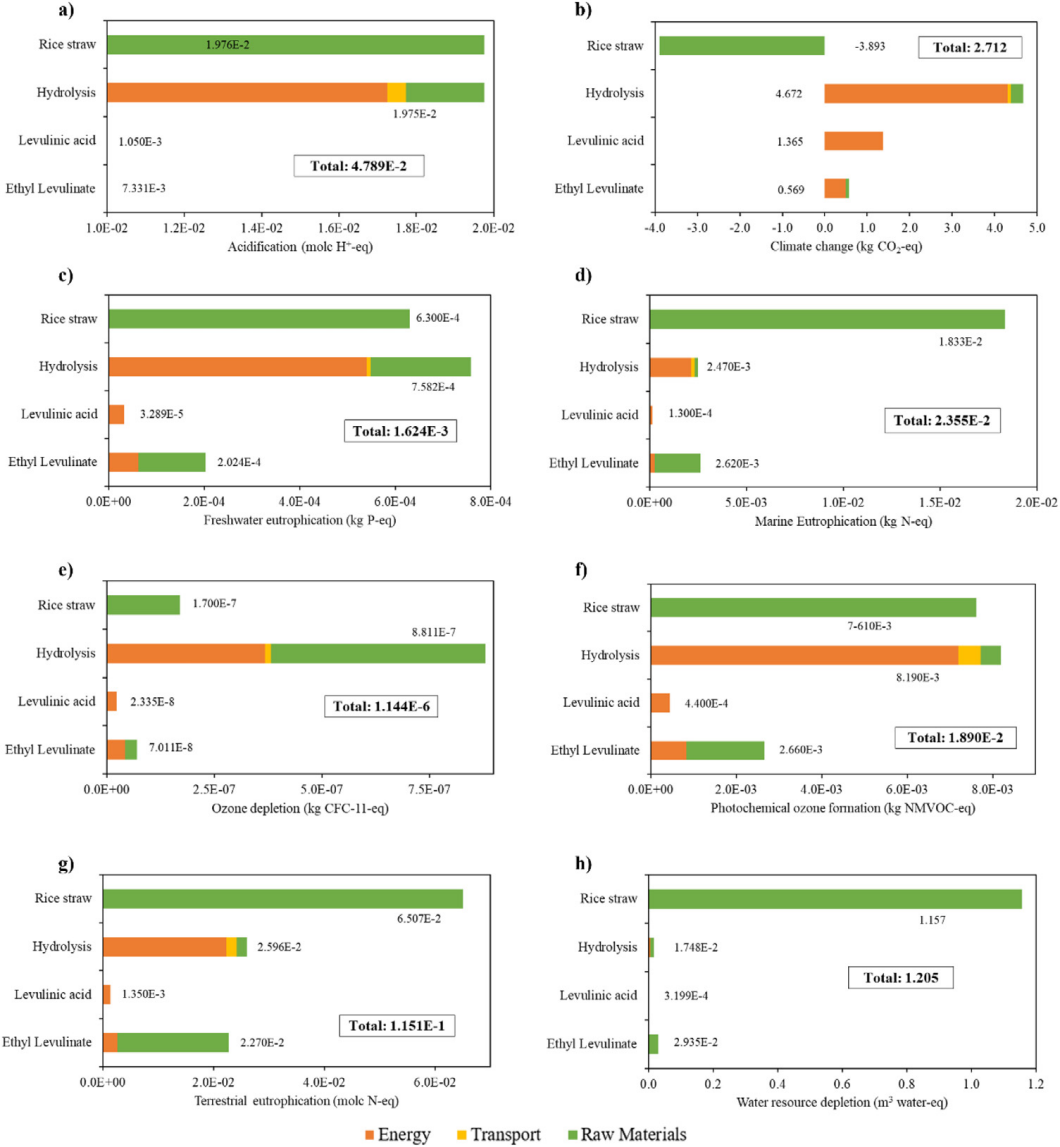
Contribution analysis for alternative scenario for EL production from RS using a 5.7 t/ha rice straw yield for a) acidification, b) climate change, c) freshwater eutrophication, d) marine eutrophication, e) ozone depletion, f) photochemical ozone formation, g) terrestrial eutrophication, and h) water resource depletion.

Two scenarios were assessed. On one hand, a base scenario without the combustion of solid hydrolysed residue corresponding to the hierarchy block HEAT-GEN (Fig. 6) and using a paddy rice yield of 4.95 t/ha. The mass and energy balance for base scenario are presented in Table 8. LCI of the base scenario is presented in Table 9. The contribution analysis performed for the base scenario is presented in Fig. 12. On the other hand, the alternative scenario includes the combustion of solid waste along with an increment of the paddy rice yield to 5.7 t/ha. The mass and energy balance for the alternative scenario is presented Table 10. LCI for alternative scenario is presented in Table 11. The contribution analysis for the alternative scenario is presented in Fig. 13.

**Table 8:** Mass and energy balance of the production of EL from rice straw in base scenario.

Stream	Kind of stream	Unit	Value
Rice straw (10 wt.% humidity)	Input	kg/h	2,000
Ethanol (99 wt.%)	Input	kg/h	108.9
Sulfuric acid (80 wt.%)	Input	kg/h	482.4
Aqueous sodium hydroxide (50 wt.%)	Input	kg/h	623.3
Water	Input	kg/h	1,601.2
Levulinic acid (98.7 wt.%)	By-product	kg/h	32.8
Furfural (99.4 wt. %)	By-product	kg/h	226.7
Sodium sulfate (98.2 wt. %)	By-product	kg/h	563.1
Ethyl Levulinate (99.5 wt. %)	Main product	kg/h	230.5
Energy consumption	Energy input	GJ/h	31.0
Electricity	Energy input	MJ/h	7.3
Cooling water	Cooling input	t/h	62.5
Air	Cooling input	t/h	5,173.4

**Table 9:** Life Cycle Inventory for base scenario (process without Heat Generation).

Process	Component	Stream type	Unit	Amount
Hydrolysis	Air	Elementary flows/Resource/in air	kg	4,941,798.000
Hydrolysis	Electricity, medium voltage	Input	kWh	1.740
Hydrolysis	Heat, from steam, in chemical industry	Input	kWh	3,956.680
Hydrolysis	Rice straw	Input	kg	2,000.000
Hydrolysis	Sodium hydroxide, without water, in 50% solution state	Input	kg	311.660
Hydrolysis	Sulfuric acid	Input	kg	385.960
Hydrolysis	Transport, freight, lorry 3.5-7.5 metric ton, EURO3	Input	kg*km	60,000.000
Hydrolysis	Water, unspecified natural origin, CO	Elementary flows/Resource	m^3^	1.606
Hydrolysis	Liquid hydrolyzed	Output	kg	8,791.000
Hydrolysis	Sodium sulfate, anhydrite	Output	kg	563.085
Hydrolysis	Solid hydrolyzed	Output	kg	1,107.000
Production of LA	Air	Input	kg	209,016.000
Production of LA	Electricity, medium voltage	Input	kWh	0.019
Production of LA	Heat, from steam, in chemical industry	Input	kWh	4,344.776
Production of LA	Liquid hydrolyzed	Input	kg	8,791.000
Production of LA	Water, cooling, unspecified natural origin, CO	Input	m^3^	260.575
Production of LA	Acetic acid	Elementary flows/Emission to water/unspecified	kg	4.180
Production of LA	Formic acid	Elementary flows/Emission to water/fresh water	kg	89.570
Production of LA	Furfural	Elementary flows/Emission to water/ground water	kg	11.760
Production of LA	Furfural, 98.5 wt.%	Output	kg	226.683
Production of LA	Glucose	Unmapped flows/water specified	kg	102.630
Production of LA	Levulinic acid	Output	kg	221.103
Production of LA	Wastewater/m3	Emission to water	m^3^	2.342
Production of EL	Air	Input	kg	22,623.000
Production of EL	Electricity, medium voltage	Input	kWh	0.259
Production of EL	Ethanol, without water, in 99.7% solution state, from fermentation	Input	kg	108.951
Production of EL	Heat, from steam, in chemical industry	Input	kWh	315.885
Production of EL	Levulinic acid	Input	kg	221.103
Production of EL	Water, cooling, unspecified natural origin, CO	Input	m^3^	53.177
Production of EL	Zeolite, powder	Input	kg	0.001
Production of EL	Ethanol	Emission to water	kg	33.590
Production of EL	Ethyl levulinate	Output	kg	230.489
Production of EL	Furfural	Emission to water	kg	0.100
Production of EL	Levulinic acid	Output	kg	32.810
Production of EL	Sulfuric acid	Emission to water	kg	0.015
Production of EL	Water, CO	Emission to water	m^3^	0.030

**Table 10:** Mass and energy balance of the production of EL from rice straw in alternative scenario.

Stream	Kind of stream	Unit	Value
Rice straw (10 wt.% humidity)	Input	kg/h	2,000
Ethanol (99 wt.%)	Input	kg/h	109.0
Sulfuric acid (80 wt.%)	Input	kg/h	473.0
Aqueous sodium hydroxide (50 wt.%)	Input	kg/h	611.1
Water	Input	kg/h	1,862.2
Lower pressure steam (125°C)	By-product	kg/h	3,100
Medium pressure steam (175°C)	By-product	kg/h	2,500
Levulinic acid (98.7 wt.%)	By-product	kg/h	32.8
Furfural (99.4 wt. %)	By-product	kg/h	226.7
Sodium sulfate (98.2wt. %)	By-product	kg/h	552.2
Ethyl Levulinate (99.5 wt. %)	Main product	kg/h	230.7
Energy consumption	Energy input	GJ/h	11.6
Electricity	Energy input	MJ/h	7.2
Cooling water	Cooling input	t/h	63.2
Air	Cooling input	t/h	5,229.6

**Table 11:** Life Cycle Inventory for alternative scenario (process with Heat Generation).

Process	Component	Stream type	Unit	Amount
Hydrolysis	Air	Input	kg	4,987,483.000
Hydrolysis	Heat, from steam, in chemical industry	Input	kWh	3,909.000
Hydrolysis	Rice straw	Input	kg	2,000.000
Hydrolysis	Sodium hydroxide, without water, in 50% solution state	Input	kg	311.660
Hydrolysis	Sulfuric acid	Input	kg	385.960
Hydrolysis	Transport, freight, lorry 3.5-7.5 metric ton, EURO3	Input	kg*km	60,000.000
Hydrolysis	Water, unspecified natural origin, CO	Input	m^3^	2.220
Hydrolysis	Liquid hydrolyzed	Output	kg	8,790.988
Hydrolysis	Sodium sulfate, anhydrite	Output	kg	563.090
Hydrolysis	Solid hydrolyzed	Output	kg	1,107.000
Production of LA	Air	Input	kg	219,527.000
Production of LA	Heat, from steam, in chemical industry	Input	kWh	242.000
Production of LA	Liquid hydrolyzed	Input	kg	8,790.988
Production of LA	Low Pressure Steam	Input	kg	1,537.000
Production of LA	Medium Pressure Steam	Input	kg	2,500.000
Production of LA	Water, cooling, unspecified natural origin, CO	Input	m^3^	263.728
Production of LA	Acetic acid	Emission to water	kg	4.180
Production of LA	Formic acid	Emission to water	kg	89.570
Production of LA	Furfural	Emission to water	kg	11.760
Production of LA	Furfural, 98.5 wt.%	Output	kg	227.000
Production of LA	Glucose	Unmapped flow/water, unspecified	kg	102.630
Production of LA	Levulinic acid	Output	kg	221.103
Production of LA	Wastewater/m3	Emission to water	m^3^	2.342
Production of EL	Air	Input	kg	22,611.000
Production of EL	Electricity, medium voltage	Input	kWh	0.259
Production of EL	Ethanol, without water, in 99.7% solution state, from fermentation	Input	kg	108.951
Production of EL	Heat, from steam, in chemical industry	Input	kWh	316.211
Production of EL	Levulinic acid	Input	kg	221.103
Production of EL	Water, cooling, unspecified natural origin, CO	Input	m^3^	53.122
Production of EL	Zeolite, powder	Input	kg	0.001
Production of EL	Ethanol	Emission to water	kg	33.590
Production of EL	Ethyl levulinate	Output	kg	230.489
Production of EL	Furfural	Emission to water	kg	0.100
Production of EL	Levulinic acid	Output	kg	32.810
Production of EL	Water, CO	Emission to water	m^3^	0.030
Solid Combustion	Air	Input	kg	13,000.000
Solid Combustion	Solid hydrolyzed	Input	kg	1,107.000
Solid Combustion	Water, turbine use, unspecified natural origin, CO	Input	m^3^	0.281
Solid Combustion	Carbon dioxide, biogenic	Emission to air	kg	1,663.460
Solid Combustion	Electricity	Output (Avoided product)	kWh	116.000
Solid Combustion	Low Pressure Steam	Output	kg	3,100.000
Solid Combustion	Medium Pressure Steam	Output	kg	2,500.000
Solid Combustion	Nitrogen	Emission to air	kg	9,971.000
Solid Combustion	Water, CO	Emission to water	m^3^	0.581
Solid Combustion	Wood ashes	Waste/unspecified	kg	338.030

LA = levulinic acid; EL = ethyl levulinate.

Finally, an economic assessment for the alternative scenario was performed. Table 12 shows the cost of raw materials, utilities, and the selling price of all products (i.e., FFR, LA, EL, sodium sulfate). Table 13 presents the cost distribution for all subroutines listed in Table 5. Table 14 and Table 15 portray the CAPEX and OPEX distribution.

**Table 12:** Cost of purchase of raw materials and utilities and selling price for by-products.

Component	Type	Value	Unit	Reference
Rice straw	Input	0.025	USD/kg	[9]
Ethanol	Input	0.9355	USD/kg	[9]
Sulfuric acid	Input	0.0991	USD/kg	[9]
Sodium hydroxide	Input	0.01275	USD/kg	[10]
Water process	Utility	0.00108	USD/kg	[11]
Cooling water	Utility	0.001318	USD/kg	[11]
Electricity	Utility	0.193	USD/kWh	[12]
Medium pressure steam	Utility	0.008627	USD/kg	[9]
High pressure steam	Utility	0.01039	USD/kg	[9]
Propane (Refrigerant)	Utility	0.674	USD/kg	[9]
Air	Utility	-	-	-
Furfural	By-product	2.2	USD/kg	[13]
Levulinic acid	By-product	5.0	USD/kg	[14]
Sodium sulfate	By-product	0.095	USD/kg	[15]
Ethyl levulinate	Main product	3.0	USD/kg	[16]

**Table 13:** Cost distribution for all subroutines in the Aspen Plus process simulation.

Hierarchy Block	Subroutine Name	Equipment Cost [USD]	Installed Cost [USD]
ESTERIF	HT-302-H	$ 9,200	$ 52,800
ESTERIF	P-302	$ 4,700	$ 31,000
ESTERIF	P-301	$ 4,600	$ 30,800
ESTERIF	RS-301	$ 48,600	$ 191,100
ESTERIF	B4	$ 17,100	$ 107,400
ESTERIF	RF-301-cond	$ 11,100	$ 67,300
ESTERIF	RF-301-cond acc	$ 17,300	$ 116,500
ESTERIF	RF-301-reb	$ 18,700	$ 78,100
ESTERIF	RF-301-reflux pump	$ 5,000	$ 30,200
ESTERIF	RF-301-tower	$ 49,200	$ 219,500
ESTERIF	HT-301-H	$ 9,500	$ 66,700
ACID-HYD	RS-102	$ 88,600	$ 246,900
ACID-HYD	HT-103-C	$ 17,000	$ 86,200
ACID-HYD	H101	$ 27,200	$ 118,200
ACID-HYD	SS-102	$ 390,000	$ 579,800
ACID-HYD	HT-101-H	$ 21,400	$ 125,300
ACID-HYD	P-101	$ 16,900	$ 44,700
ACID-HYD	RS-103	$ 84,600	$ 239,500
ACID-HYD	SS-101	$ 390,000	$ 579,800
ACID-HYD	RS-101	$ 88,600	$ 249,200
ACID-HYD	HT-103-C	$ 150,600	$ 296,300
ACID-HYD	HT-102-H	$ 21,100	$ 107,900
FFR-SEP	HT-204-C	$ 17,100	$ 116,600
FFR-SEP	HT-202-C	$ 9,200	$ 50,000
FFR-SEP	RF-201-cond	$ 15,600	$ 82,500
FFR-SEP	RF-201-cond acc	$ 17,300	$ 117,700
FFR-SEP	RF-201-reb	$ 35,600	$ 118,500
FFR-SEP	RF-201-reflux pump	$ 5,000	$ 31,400
FFR-SEP	RF-201-tower	$ 119,700	$ 312,800
FFR-SEP	P-201	$ 4,400	$ 30,700
FFR-SEP	HT-201-C	$ 24,000	$ 102,800
FFR-SEP	P-303	$ 17,900	$ 52,300
FFR-SEP	M201	$ 12,100	$ 72,500
HYD-SEP	RF-110-cond	$ 8,700	$ 48,400
HYD-SEP	RF-110-cond acc	$ 17,300	$ 110,500
HYD-SEP	RF-110-reb	$ 14,000	$ 64,800
HYD-SEP	RF-110-reflux pump	$ 5,000	$ 30,200
HYD-SEP	RF-110-tower	$ 35,400	$ 192,600
HYD-SEP	FL-111-flash vessel	$ 17,100	$ 115,100
HYD-SEP	HT-111-C	$ 16,100	$ 83,000
HYD-SEP	SEP-110	$ 17,100	$ 115,100
HYD-SEP	HT-110-H	$ 18,000	$ 103,600
HYD-SEP	FL-110-flash vessel	$ 19,800	$ 119,000
HYD-SEP	HT-112-H	$ 18,100	$ 103,500
HEAT-GEN	HT-401-H	$ 20,500	$ 104,700
HEAT-GEN	TUR-401	$ 115,700	$ 285,600
HEAT-GEN	HT-403-C	$ 15,700	$ 82,600
HEAT-GEN	P-402	$ 5,300	$ 35,600
HEAT-GEN	HT-402-C	$ 12,100	$ 69,700
HEAT-GEN	HT-404-C	$ 21,000	$ 100,000
HEAT-GEN	TUR-402	$ 116,400	$ 275,900

**Table 14:** CAPEX distribution by items using Lang Factors for alternative scenario.

CAPEX	Solid-fluid processing plant	Value (USD)
Direct costs		
Purchased equipment	1	$ 2,262,200
Delivery, percent of purchased equipment	0.1	$ 226,220
Purchased equipment installation	0.39	$ 882,258
Instrumentation and controls	0.26	$ 588,172
Piping	0.31	$ 701,282
Electrical	0.1	$ 226,220
Buildings	0.29	$ 656,038
Yard improvement	0.12	$ 271,464
Service facilities	0.55	$ 1,244,210
*Total direct costs*		$ 7,058,064
		
Indirect costs		
Engineering and supervision	0.32	$ 723,904
Construction expenses	0.34	$ 769,148
Legal expenses	0.04	$ 90,488
Contractor's fees	0.19	$ 429,818
Contingency	0.37	$ 837,014
Working capital	0.75	$ 1,696,650
*Total indirect costs*		$ 4,547,022
	**CAPEX**	**$ 11,605,086**

**Table 15:** OPEX distribution by item using Lang Factors for alternative scenario.

O & M costs (USD/year)	
Variable production costs	Value (USD)
Raw materials	$ 1,542,598
Operating labor	$ 885,177
Direct supervisory and clerical labor	$ 88,518
Utilities	$ 1,770,354
Maintenance and repairs	$ 580,254
Operating supplies	$ 116,051
Laboratory charges	$ 88,518
Patents and royalties	$ 354,071
*Total variable production costs*	$ 5,425,540
	
Fixed charges	
Local taxes	$ 464,203
Insurance	$ 116,051
Rent	$ 696,305
Financing (interest)	$ 290,127
*Total fixed charges*	$ 1,566,687
	
Plant overhead costs	$ 1,062,212
	
General expenses	
Administrative costs	$ 265,553
Distribution and marketing costs	$ 885,177
Research and development costs	$ 442,589
*Total general expenses*	$ 1,593,319
**OPEX (USD/year)**	**$ 9,647,758**

## 3 Experimental Design, Materials and Methods

The purpose of this document is to gather all the relevant information to calculate the LCI to carry out the LCA to produce EL from RS, as shown in the main manuscript. Table 1 shows the methodology to calculate the LCI of each stage involved in the conversion of RS into EL. Detailed information is shown in the upcoming sections.

## 4 Life Cycle Inventory

### 4.1 Rice straw production

LCI for the production of RS was obtained for Colombia conditions, in the Orinoquia region [2]. RS production encompasses both cultivation and harvest stages. An average paddy rice yield of 4.97 t/ha was used for inventory calculation [3]. Diesel, fertilizers, land resources, and energy were the main inputs. Emissions to air and water were also considered. Paddy rice and RS were considered as main outputs. Diesel, land, and fertilizer requirements for rice production was obtained from the literature review [2]. Emissions of the agricultural stage was determined according to the IPCC methodology. Emissions associated with the use of nitrogen fertilizers included NH_3,_ NO_x_, N_2_O, and NO_3_. The latter was calculated according to the SQCB (Sustainable Quick Check for Biofuels) methodology. Phosphorous emissions to water were calculated using the SALCA (Swiss Agricultural Life Cycle Assessment) model [4]. Emissions associated with diesel combustion in the agroindustry were calculated based on the emissions factors reported by Martinez-Gonzales et al. [5]. CH_4_ emission associated with rice cultivation was also included. Carbon sequestered by the crop was calculated in terms of the proximate analysis rice and the carbon content of each fraction as shown in Table 2 reported in literature [6]. Rainfall and irrigation needs were calculated using the TURC methodology. The LCI for paddy rice culture in Orinoquia region in Colombia is present in Table 3.

### 4.2 Rice straw valorization to EL

Fig. 1 shows a complete process to produce EL from RS was developed in Aspen Plus v.12 (Aspen Tech, MA, USA) by considering four main stages: (i) RS acid hydrolysis; (ii) purification of LA; (iii) purification of FFR; and (iv) production and purification of EL. The Non-Random Two-Liquid with Redlich-Kwon (NRTL-RK) was used as the main thermodynamic package for phase equilibrium and thermodynamic estimations. However, due to the scarcity of some binary parameters for modelling the equilibrium phase with 3,5-hydroxymethylfurfural (HMF) and other lignocellulosic by-products (e.g., FFR, LA, formic acid (FA) and acetic acid (AA)), the Dortmund modified UNIFAC (UNIFAC-DMD) group contribution was employed to calculate the activity coefficients. The UNIFAC-DMD provides more reliable behavior of the phase equilibria of compounds than the traditional UNIFAC method. RS was modelled in terms of cellulose (38.3 wt.%), hemicellulose (28 wt.%), lignin (14.9 wt.%), and ashes (18.8 wt.%) based on data presented in Table 2.

The three former was modelled according to the properties shown by Wooley and Putsche [7]. Whereas ashes were modelled as dioxide silicon due to this is the main constitutive element in this biomass fraction. Humins were modelled as cellulose and hemicellulose since those are decomposition products during the acid hydrolysis of biomass. Auxiliary units such as heat exchangers, pumps, compressors, valves, mixers, and splitters were considered within the simulation of the overall process. Kinetics models for reactions were not considered due to the main objective with the simulation is to obtain mass and energy balances for life cycle inventory purposes and the sizing or design of the equipment is not part of the study scope.

Table 4 shows a description of main subroutines using in the process simulated with Aspen Plus v12. A brief description, as well as the operating conditions and assumptions used for each unit are presented. And Table 5 present the purpose of each subroutine employed among the simulation in Aspen Plus v12. Mixers and Flow Splitters were not considered for data presented in Table 4 and Table 5.

### 4.3 Rice straw pretreatment

Fig. 2 presents the detailed diluted acid hydrolysis flowsheet, which corresponds to the Hierarchy block name ACID-HYD shown in Fig. 1. Herein, steam explosion and diluted acid hydrolysis were employed together as pretreatment method to pretreat RS. Steam explosion was used as alternative to remove the hemicellulose fibers and ease the hydrolysis of hemicellulose and cellulose. In this first reaction stage, sulfuric acid was employed based on Biofine process at 210°C and 20 bar. A second diluted acid hydrolysis with sulfuric acid was employed since its widely used at industrial level to pretreat lignocellulosic biomass at 190°C and 18 bar. Table 6 shows the conversion rates of main reactions during the acid hydrolysis of RS and LA esterification based on the literature review. Aside from the acid hydrolysis stage, the pretreatment also includes the neutralization of sulfuric acid with sodium hydroxide, the recovery of unreacted cellulose, hemicellulose, lignin, and humins, hereafter names as HYD-SOL. Also includes the recovery of sodium sulfate as by-product for sale. All reactors employed to pretreat the biomass were RStoic subroutine. The heat generated in the first reaction stage was used to preheat the process water used for dilute acid hydrolysis. The liquid to solid ratio employed for dilute acid hydrolysis was 8:1, using a water recycle equivalent to 77% of the water used as solvent in the hydrolysis reaction. Two solid separation units were employed to separate the unreacted sugars and the sodium sulfate produced in the neutralization reactor (RS-103).

### 4.4 Levulinic acid and furfural purification

Fig. 3 (LA production) present the detailed flowsheet for LA purification. Distillation column was modelled using a RadFrac module with *Strongly non-ideal liquid* convergence method. The purification of LA was designed based on the literature review, where a distillation train formed by two flash separators and a distillation column was used. Flash separators were modelled using Flash-2 module, and the heat remanent from the vapor phase of the first flash unit was used to reheat the liquid phase feed to the second flash unit. The flash units were modeled as adiabatic units and no pressure drop inside de vessel. Temperature and pressure conditions required for each flash were set using a heat exchanger and a valve before each flash feed.

Fig. 4 (FFR purification) presents the detailed flowsheet for FFR purification. The FFR purification was carried out using azeotropic distillation based on Zeitsch [8]. Azeotropic distillation columns were also modelled with RadFrac module but using the *Azeotropic* convergence method. Efficiency of distillation columns were adjusted to 65% according to heuristics rules. A decanter was used to separate the distillate from the first distillation tower into a furfural-rich phase, which was subsequently distilled to obtain a high purity FFR stream and the removal of volatiles such as formic acid (FA) and the remaining water in the distillate. The aqueous rich phase from decanter DC-201 was recirculated to the azeotropic column to maximize FFR recovery. The bottom stream from RF-201 (azeotropic column) was recycled to the dilute acid hydrolysis stage to reduce the total water consumption of the overall process and to minimize the FFR lost in the two-step separation train.

### 4.5 Ethyl levulinate production

Fig. 5 presents the detailed flowsheet for EL production. This was done through the esterification of LA with ethanol using a desilicated DH-ZSM-5 zeolite as catalyst with a catalyst load of 13wt%. Esterification was modelled with a RStoic unit at 120°C and 4.5 bar, using ethanol as solvent (8:1 ratio) with a conversion of LA of 85% as shown in Table 6. Kinetic model for this reaction was not considered due to the simulation purpose is not the equipment design or sizing instead the mass and energy balances calculation for LCI. Purification of EL was performed by using a distillation train where RadFrac modules with *Strongly non-ideal liquid* convergence method were employed to module the two distillation columns. Efficiency of distillation columns were adjusted to 65% according to heuristics rules. In the first column, a distillate rich in ethanol and water is obtained, which is subjected to a separation process with molecular sieves for the recovery and recirculation of 95% of the remaining ethanol using a SEP-2 unit. The bottom stream, containing LA and EL is fed to a second distillation tower, where two high purity streams are obtained, one of EL and the other of LA.

### 4.6 Heat integration

The integration consisted of using the remaining heat from the first acid hydrolysis reactor and the output stream of the second acid hydrolysis reactor to reduce the energy consumption associated with the generation of the steam required for hydrolysis, according to Fig. 2**.** Likewise, the two steam streams obtained from the flash units associated with the separation of the FFR and the LA were integrated for heating the liquid hydrolysate entering the separation train of the HYD-SEP hierarchy, according to Fig. 3 (HYD-SEP) and Fig. 4 (FFR-SEP). The other streams were not integrated because their flows were too small, and the temperature differences did not satisfy the minimum temperature approach value of 10°C. The MHeatX unit was employed to model de heat exchangers associated with the heat integration implementation.

### 4.7 Combustion of solid hydrolyzed residue

Fig. 6 presents the detailed flowsheet for LPS and MPS generation. RStoic unit was used to model combustion by stoichiometric reactions of solid waste combustion assuming complete combustion and disregarding the generation of methane, NOx, and SOx. The combustion temperature was set in 904°C at a pressure of 10 bar. Excess air was used to control the reactor temperature.

The condenser was modeled using a Heater unit cooled by air and the boiler was modeled using a non-rigorous MHeatX unit. Ashes from the combustion unit was separated from the reactor outlet stream using an SEP-2 unit. Two isentropic turbines were used to generate the MPS (saturated steam at 175°C) and the LPS (125°C). A total of 5,600 kg/h of water was used to generate 3,100 kg/h of LPS (100% of the requirement of the process) and 2,500 kg/h of MPS (55% of the total process requirement). Both LPS and MPS generated was recirculated to the generation cycle, reducing the water consumption to a make-up stream of 280 kg/h of water.

### 4.8 Sensitivity analysis for distillation columns design

A sensitivity analysis was performed to determine the best operation conditions for the five distillation columns used among the process. The number of equilibrium stages, optimal feed stage, reflux molar ratio, and distillation or bottom to feed ratio were assessed to minimize the energy requirements in the distillation column and maximize the recovery of the interest product.

Table 7 presents the operation conditions selected for each distillation column. Detailed sensitivity analysis is shown in Fig. 7 (RF-110), Fig. 8 (RF-201), Fig. 9 (RF-202), Fig. 10 (RF-301), and Fig. 11 (RF-302). The operation pressures were selected seeking the reduction of reboiler and condenser duty requirement.

### 4.9 Mass and energy balance, Life Cycle Inventory, and Contribution Analysis

Mass and energy balance were retrieved from the simulation results in Aspen Plus. The Life Cycle Inventory (LCI) was estimated using the Aspen Plus balances and the Ecoinvent Database for background data. Contribution analysis was performed in Open LCA v 1.10 software using the ILCD 2011 midpoint + baseline method with eight impact categories assessed: (1) Acidification (molc H+-eq); (2) Climate Change (kg CO_2_-eq); (3) Freshwater Eutrophication (kg P-eq); (4) Marine Eutrophication (kg N-eq); (5) Ozone Depletion (kg CFC-11-eq); (6) Photochemical Ozone Formation (kg NMVOC-eq); (7) Terrestrial Eutrophication (molc N-eq); and (8) Water Resource Depletion (m^3^ water-eq).

### 4.10 Base scenario

Table 8 presents the mass and energy balance to produce EL from RS in the base scenario. Fig. 12 presents the contribution analysis of all impact categories described above in the base scenario. Finally,

Table 9 presents the LCI of the production of EL from RS in the base scenario.

### 4.11 Alternative scenario

Table 10 presents the mass and energy balance to produce EL from RS in the alternative scenario and Table 11 presents the LCI of the production of EL from RS in the alternative scenario. Finally, Fig. 13 shows the LCI of the production of EL from RS using a paddy rice yield of 5.7 t/ha.

### 4.12 Economic assessment

An economic assessment for the alternative scenario was performed to give insights into the feasibility of the process in an integrated way. Table 12 presents the cost of raw materials, products, by-products, and co-products retrieved from the literature review and used for calculation of economic indicators.

Table 13 presents the equipment cost and installation cost, obtained from Aspen Plus Economic Analyzer and used for CAPEX calculation. Table 14 depicts the CAPEX distribution estimated using Lang-Factors and Table 15 present the OPEX distribution using Lang-Factors.

## Ethics Statement

This work did not involve human subjects or laboratory animal, therefore did not meet any ethical issues.

## CRediT Author Statement

**Cristhian Cañon:** Writing – original draft preparation, Visualization, Investigation, Process Simulation; **Nestor Sanchez:** Conceptualization, Methodology, Writing – review & editing; **Martha Cobo:** Conceptualization, Methodology, Supervision, Writing – review & editing, Project administration, Funding acquisition.

## Declaration of Competing Interest

The authors declare that they have no known competing financial interests or personal relationships which have or could be perceived to have influenced the work reported in this article.

## Data Availability

Ethyl_Levulinate_from_Colombian_Rice_Straw (Original data) (Mendeley Data) Life Cycle Inventory data for ethyl levulinate production from Colombian rice straw (Original data) (Data in brief).
